# Genetic components of stringent response in *Vibrio cholerae*

**Published:** 2011-02

**Authors:** Ritesh Ranjan Pal, Bhabatosh Das, Shreya Dasgupta, Rupak K. Bhadra

**Affiliations:** *Infectious Diseases & Immunology Division, Indian Institute of Chemical Biology (CSIR), Kolkata, India*; * *Present address*: Centre de Genetique Moleculaire, Batiment 26, CNRS UPR2167, Avenue de la Terrasse 91198, Gif Sur Yvette, France

**Keywords:** *cgtA*, *dksA*, (p)ppGpp, *relA*, *spoT*, stringent response, *Vibrio cholerae*

## Abstract

Nutritional stress elicits stringent response in bacteria involving modulation of expression of several genes. This is mainly triggered by the intracellular accumulation of two small molecules, namely, guanosine 3’-diphosphate 5’-triphosphate and guanosine 3’,5’-bis(diphosphate), collectively called (p)ppGpp. Like in other Gram-negative bacteria, the cellular level of (p)ppGpp is maintained in *Vibrio cholerae*, the causative bacterial pathogen of the disease cholera, by the products of two genes *relA* and *spoT*. However, apart from *relA* and *spoT*, a novel gene *relV* has recently been identified in *V. cholerae*, the product of which has been shown to be involved in (p)ppGpp synthesis under glucose or fatty acid starvation in a Δ*relA* Δ*spoT* mutant background. Furthermore, the GTP binding essential protein CgtA and a non-DNA binding transcription factor DksA also seem to play several important roles in modulating stringent response and regulation of other genes in this pathogen. The present review briefly discusses about the role of all these genes mainly in the management of stringent response in *V. cholerae*.

## Introduction

Bacteria have immense capability to modulate their gene expression according to various environmental conditions. Among such conditions, nutrient limitation is a critical factor that determines their survival and growth. The adaptive response to nutritional stress in microbial cells leads to rapid and complex metabolic adjustments through modulation of gene expression and regulation, which is widely known as the stringent response^1^. Being an environmental pathogen, *Vibrio cholerae*, that causes severe diarrhoeal disease cholera, faces several stresses including nutritional scarcity while staying in aquatic bodies as well as in host gastrointestinal tract. In order to survive and grow, the pathogen must sense and adapt to these frequent changes of various factors in their surrounding environment. Thus, the stringent response may play a critical role in survival of this microorganism under stress conditions. The abrupt global changes in gene expression associated with the stringent response are mainly triggered by the intracellular accumulation of two small molecules called guanosine 3’-diphosphate 5’-triphosphate and guanosine 3’,5’-bis(diphosphate), collectively called (p)ppGpp^1^.

**Fig. 1:**
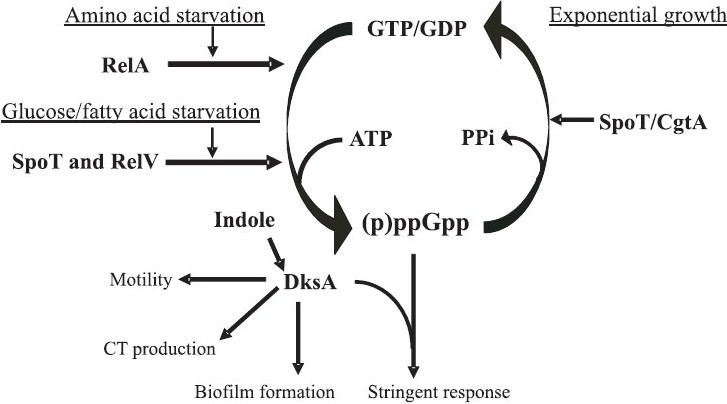
Schematic diagram showing involvement of multiple enzymes/proteins in regulation of stringent response and other functions in *V. cholerae*. During amino acid starvation RelA is activated and synthesizes (p)ppGpp, while fatty acid or glucose starvation induces activation of SpoT and RelV, which also synthesize (p)ppGpp. (p)ppGpp with the help of DksA induces stringent response.

Generally, in Gram-negative γ-proteobacteria the cytoplasmic level of (p)ppGpp is maintained by the products of two genes *relA* and *spoT*, called RelA and SpoT, respectively. While (p)ppGpp is synthesized by the RelA enzyme under amino acid starved condition, SpoT hydrolyses it^1^. However, SpoT is a bifunctional enzyme with weak (p)ppGpp synthesizing activity that can synthesize (p)ppGpp under glucose or fatty acid starved condition^1^ (Fig. 1). The biology of stringent response is extensively studied in *Escherichia coli*. However, recent studies on *V. cholerae* and also in other bacteria indicate that apart from canonical RelA/SpoT, these organisms may code for novel (p)ppGpp synthetases. Further, recent studies indicate that an RNA polymerase-associated small regulatory protein, called DksA, and a conserved essential GTP binding protein CgtA are also involved in regulation of stringent response by modulating the activity of (p)ppGpp^1^. Since the RelA and SpoT enzymes of *V. cholerae* are structurally and functionally very similar to that of *E. coli*, the present review will primarily focus on RelV, DksA and CgtA proteins.

## The *relA* and *spoT* genes of *V. cholerae*

Haralalka *et al*^2^ first demonstrated that like in *E. coli*, *V. cholerae* *relA* gene product is involved in (p)ppGpp synthesis. This is supported by the fact that *V. cholerae* *relA* null mutant cells failed to accumulate (p)ppGpp upon amino acid starvation. In *E. coli*, it has been shown that when the rate of tRNA aminoacylation does not meet the demands of protein synthesis, the ribosome associated *relA* is activated^1^. The activated *relA* then triggers the stringent response through conversion of GTP to (p)ppGpp, which finally leads to the rapid inhibition of syntheses of stable RNAs, ribosomes, and proteins, and ultimately arrest of cell growth. It is expected that a similar mechanism is operative in *V. cholerae* cells. However, unlike *E. coli*, *V. cholerae* *relA* mutant showed severe growth defect in M9 minimal (MM) medium lacking amino acids^2^,^3^. It is known that wild type *E. coli* cells can grow in nutritionally poor MM medium due to synthesis of (p)ppGpp, which promotes amino acid biosynthesis operons. Thus, the growth of *E. coli relA* mutant in M9 medium has been explained due to the (p)ppGpp synthetic activity of SpoT^4^. It is to be noted that like *E. coli*, *V. cholerae* also possesses the *spoT* gene and Das *et al*^5^ showed that the *spoT* gene is functional in *V. cholerae*. The growth defect of *V. cholerae* *relA* mutant in MM medium even in the presence of functional *spoT* gene could be due to activation of a gene, which codes for a novel (p)ppGpp synthetase. It appears that, in the absence of RelA, this novel (p)ppGpp synthetase most likely produces a large quantity of (p)ppGpp, which is toxic to cells.

Both RelA and SpoT proteins have similar multi-domain structures and the domains from N- terminus to C-terminus are: HD [(p)ppGpp hydrolase], Rel-Spo [(p)ppGpp synthetase], TGS (named after threonyl-tRNA synthetase, GTPases and SpoT proteins where this domain is conserved) and ACT (named after acetolactate synthetase, chorismate mutase and TyrR proteins where this domain is conserved)^1^,^5^. Das *et al*^5^ have demonstrated that the ACT domain of SpoT protein of *V. cholerae* is essential for (p)ppGpp hydrolase activity. Since *E. coli* SpoT has a very similar structure, it is expected that the ACT domain may play similar function in *E. coli*. Battesti and Bouveret^6^ have shown that during fatty acid starvation (p)ppGpp synthetase activity of SpoT is regulated through an interaction of the TGS domain of the enzyme with the acyl carrier protein (ACP), a central co-factor in fatty acid biosynthesis.

## Discovery of RelV

While characterizing *V. cholerae* Δ*relA* Δ*spoT* mutants, Das and Bhadra^7^ provided evidence that in *V. cholerae* there is a probable third source of (p)ppGpp synthesis. When they tried to develop a (p)ppGpp^0^ strain by deleting *relA* and *spoT*, the double mutant still produced sufficient (p)ppGpp under glucose or fatty acid starved condition^5^,^7^. In contrast to this observation, *E. coli* Δ*relA* Δ*spoT* mutant is phenotypically a (p)ppGpp^0^ strain^1^. Ultimately, Das *et al*^5^ were able to identify the gene *relV* and showed that apart from *relA* and *spoT*, *relV* is also responsible for the production of (p)ppGpp (Fig. 1). In fact, *relV* is highly conserved in *Vibrio* spp^5^. Unlike RelA/SpoT, RelV is a small protein with a single domain of Rel-Spo. It is interesting to note that genes, which code for similar proteins are also present in the Gram-positive organisms such as *Bacillus subtilis* and *Streptococcus mutans*. However, unlike Gram-negatives, the Gram-positive bacteria possess a single gene *rel*, which codes for an enzyme Rel having both (p)ppGpp synthetase as well as hydrolase activity. Discovery of *relV*-like gene in certain bacteria raised several questions such as like (*i*) how the gene is regulated, (*ii*) under what circumstances the gene is expressed, (*iii*) what are the signals that induces expression of the gene, and (*iv*) what is the role of the gene in maintaining basal level of (p)ppGpp? Furthermore, deletion of *relA* and spoT leads to slow growth of *V. cholerae* cells during lag to log phase in a nutritionally rich medium and the defect is corrected in a *relA* *spoT relV* triple mutant indicating that *relV* is most likely activated in early log phase and leads to high basal level of (p)ppGpp, which is inhibitory to growth^5^. However, at present it is not clear how the *relA spoT* mutant cells manage to degrade (p)ppGpp in the absence of (p)ppGpp hydrolase enzyme SpoT.

## CgtA and stringent response

CgtA is a highly conserved essential GTP-binding protein, coded by the *cgtA* gene, belonging to Obg subfamily in prokaryotes^8^. CgtA has been suggested to play important roles in various physiological processes, *e.g*., regulation of initiation of sporulation, DNA replication, chromosome partitioning, replication fork stability, chromosome segregation, ribosome maturation, *etc*. Recent studies indicate that CgtA is involved in maintaining steady-state level of (p)ppGpp during exponential growth^9^. CgtA was found to be associated with the 50S ribosomal subunit^10^. Crystal structure analysis of the full-length CgtA/Obg protein from *Thermus thermophilus* revealed three domains; a central GTP-binding domain flanked by N- and C-terminal domains designated OBG and OCT, respectively that are unique to the Obg protein^11^. Buglino *et al*^12^ showed that (p)ppGpp is bound with G domain in the crystal structure of C-terminally truncated Obg/CgtA protein of *B. subtilis* and proposed that CgtA/Obg probably recognizes (p)ppGpp in response to starvation or stress. Reports suggest the role of CgtA in stringent response since it has been co-purified with the SpoT protein in *E. coli*^10^. In the case of *V. cholerae*, CgtA is an essential protein and has been shown to interact with SpoT^9^,^13^. It has been suggested that CgtA most likely modulates the SpoT function for proper maintenance of cellular (p)ppGpp level^9^ (Fig. 1).

## DksA, a pleiotropic regulator

**Fig. 2:**
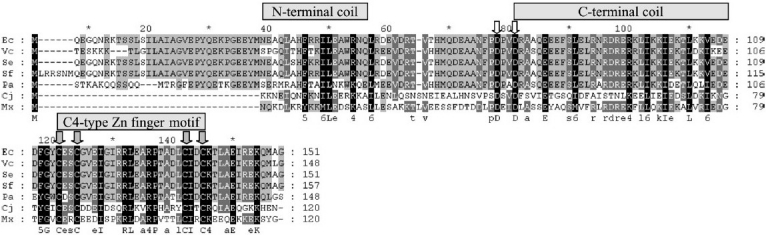
Amino acid sequence alignment (GeneDoc software) of *dksA* homologues of several Gram-negative bacteria, namely, *E. coli* (Ec), *V. cholerae* (Vc), *S. enterica* serovar Typhimurium (Se), *S. flexneri* (Sf), *P. aeruginosa* (Pa), *C. jejuni* (Cj) and *M. xanthus* (Mx). Amino acid residue numbers of each *dksA* homologue are shown in the right margin. Dark and light shadings indicate identical or similar residues, respectively. Coiled-coil domain consists of N-terminal and C-terminal coils as indicated. C4-type Zn finger motif present in the C-terminal part of *dksA* is also shown. Two highly conserved aspartic acid (D) residues of the tip of coiled-coil domain of *dksA* have been proposed to be critical for co-ordinating (p)ppGpp bound Mg^2+^ with the RNA polymerase secondary channel and are indicated by hollow vertical arrows. Four highly conserved cysteine (C) residues of C4-type Zn finger motif are also indicated by grey vertical arrows.

The *dksA* gene is highly conserved in Gram-negative bacteria including in *V. cholerae*. From bioinformatic analysis it appears that the *dksA* gene of *V. cholerae* codes for a small protein of ~ 17-kDa in size, which is similar to that of *E. coli* DksA protein. However, unlike *cgtA*, *dksA* is not an essential gene in *V. cholerae*, since *V. cholerae* Δ*dksA* mutant is viable (unpublished observation). This is similar in other bacteria including *E. coli*, except in *Myxococcus xanthus* where *dksA* has been reported to be an essential gene^14^. The *dksA* gene function was first identified as a dose-dependent multicopy suppressor of temperature sensitivity and filamentation phenotype of a *dnaK* gene deleted mutant of *E. coli*^15^. Conservation of *dksA* in microbial genomes suggests that the product of this gene most likely play important roles in bacterial physiology. The 2.0 Å resolution structure of *E. coli* *dksA* has been reported^16^, which revealed a globular domain and coiled coil (consists of two long N- and C-terminal α-helices) structure of the protein. Amino acid sequence alignment of DksA homologues of various Gram-negative bacteria including the dksA protein of *V. cholerae* indicates that *dksA* is well conserved, especially its C-terminal half, which contains a potential coiled coil motif with several invariant amino acid residues and a C4-type Zn finger motif^16^ (Fig. 2).

DksA has pleiotropic functions in bacteria, which include modulation of stringent response^16^–^18^, gene expression^19^,^20^, quorum sensing^20^ and pathogenesis^21^–^25^. Among these, modulation of functions of stringent response appears to be important. Several studies have suggested that DksA acts as a co-regulator for the (p)ppGpp-dependent regulation of genes. A *dksA* mutant strain of *E. coli* showed auxotrophy that can be suppressed by a *rpoB* mutation (βT563P), which also suppresses the auxotrophy of a (p)ppGpp negative strain, called (p)ppGpp^0^ strain^17^. The effect of *dksA* mutation on induction of the *rpoS* gene expression is very similar to that observed in a (p)ppGpp^0^ strain^26^. It has also been shown that DksA enhances the effect of (p)ppGpp on both negatively and positively regulated genes under *in vivo* as well as *in vitro* conditions^18^. However, further comparative analysis of the phenotypes of Δ*dksA* and (p)ppGpp^0^ strains allowed to identify some differential phenotypes, for example, amino acid requirements are not exactly the same for these genetically defined strains and the auxotrophic phenotype of the (p)ppGpp^0^ strain cannot be restored by over-expressing DksA. Adhesion, chemotaxis and motility phenotypes are also quite different in the case of a Δ*dksA* mutant versus a (p)ppGpp^0^ strain^27^. It has been shown that DksA and (p)ppGpp may exert independent effects on gene transcription under both *in vivo* and *in vitro* situations^27^.

DksA belongs to a unusual family of transcriptional regulators which does not bind directly to the regulatory part of a gene, rather it binds directly to the secondary channel of RNA polymerase (RNAP) as shown in the case of *E. coli*^1^,^16^. When DksA binds directly to RNAP, two highly conserved aspartic acid residues of the protein (Fig. 2) help in stabilizing the (p)ppGpp-Mg^2+^ -RNAP complex^16^. DksA decreases the half-life of the open complexes formed upon transcription initiation and amplifies the (p)ppGpp effect^16^,^18^. The hallmark of the stringent response is downregulation of stable RNA synthesis (rRNAs and tRNAs) and ribosome production under amino acid starvation. In addition to downregulation of stable RNA synthesis, transcription from a number of promoters that control the expression of genes involved in survival and stress adaptation, are also stimulated during stringent response. Not only negative effects of (p)ppGpp are amplified by DksA, the positive influence of DksA in transcription from amino acid biosynthesis gene promoters have also been reported^18^,^24^. Thus, it appears that DksA functions as a co-factor for (p)ppGpp by synergistically amplifying the effect of (p)ppGpp depending on specificity of promoters.

Apart from its involvement in stringent response, several studies also indicate that DksA may regulate flagella synthesis in motile bacteria^27^,^28^. The ability of bacteria to swim with the help of flagella toward or away from specific environmental stimuli, such as nutrients, oxygen, or obnoxious substances provides cells with a survival advantage, especially under nutrient-limiting conditions. Flagella synthesis during stationary phase and after starvation was found to be inhibited by DksA and (p)ppGpp. This role of DksA not only co-ordinates ribosome assembly and flagella synthesis but also prevents expenditure of limited energy resources on two of the cell’s intense energy demanding processes of macromolecular synthesis^28^. From mutational studies, it appears that DksA is involved in motility of *V. cholera*^29^ (Fig. 1), an important phenotype of this human pathogen, which is considered important for pathogenesis.

A growing number of recent studies indicate involvement of DksA in processes related to growth, stress, starvation, and survival that eventually affect the pathogenic potential of a bacterium. A common scenario is that when DksA is absent, pathogenicity is compromised for reasons that vary with the organism studied. Inhibitory effects can also occur on host interactions that enhance pathogen survival, invasiveness, or persistence. Examples include *Salmonella enterica* serovar Typhimurium, *Shigella flexneri, Pseudomonas aeruginosa, Campylobacter jejuni, E. coli* and *V. cholerae*. In *S. enterica* serovar Typhimurium, *dksA* gene product controls the expression of the stationary phase sigma factor *rpoS* (σ^38^) and as a result *dksA* mutant was less virulent than the parental strain when tested in mice as well as in 3-week-old hatched chickens^25^. In *S. flexneri*, DksA is involved in intracellular spread upon infection of epithelial cell layers^24^. Unlike in *Salmonella*, the effect of DksA does not depend on the sigma factor RpoS in *S. flexneri*^22^. In *P. aeruginosa*, the *las* and rhl quorum sensing systems control the secretion of extracellular virulence factors including rhamnolipids and LasB elastase^21^. DksA is also involved in post-transcriptional control of the extracellular virulence factor production in *P. aeruginosa*. In *C. jejuni, dksA*-like protein exhibited a decreased ability to invade intestinal cells and induced release of interleukin-8 from intestinal cells^20^. Similarly, it has been reported that adherence capacity of enterohaemorrhagic *E. coli* and gene expression in the locus of enterocyte effacement are modulated by (p)ppGpp and DksA^23^. Interestingly, in *V. cholerae* significant reduction of cholera toxin production, its principal virulence factor was observed under *in vitro* condition^29^. Recently, Mueller *et al*^30^ reported that mutation of *dksA* is probably responsible for indole non-responsive phenotype in *V. cholerae* (Fig. 1). Indole is a natural breakdown product of the tryptophan and it can act as a stationary phase signal molecule that induces biofilm formation. Analysis of *V. cholerae* Δ*dksA* mutants showed severe growth defect in MM medium lacking amino acids. However, unlike *E. coli* ΔdksA strain, *V. cholerae* Δ*dksA* mutants initiated growth after 5-6 h and overnight incubation allowed the culture to reach into saturation^29^(unpublished observation). Although the reason behind this is currently not clear, it could be due to excess (p)ppGpp production by the RelV protein of *V. cholerae*. It may be possible that accumulation of sublethal amount of (p)ppGpp could induce amino acid biosynthesis operons leading to growth even under *dksA* negative background. Thus, DksA also appears to be a pleiotropic regulator in *V. cholerae*, controlling stringent response, motility and virulence factor production (Fig. 1). Future studies are needed to unravel the exact regulatory functions of DksA in this important human pathogen.

## Conclusion

Recent studies indicate that multiple genes are involved in modulation of stringent response in *V. cholerae*. Participation of RelA and SpoT enzymes along with the essential GTP binding protein CgtA in subtle regulation of intracellular levels of (p)ppGpp as well as the involvement of recently discovered novel synthetase RelV indicate that (p)ppGpp metabolism is highly complex in *V. cholerae*. Further, genetic evidences indicate that, as in the case with *E. coli*, the conserved regulatory protein DksA also modulates the action of (p)ppGpp in *V. cholerae*. Apart from stringent response regulation, DksA may also be directly or indirectly involved in virulence factor production and motility of *V. cholerae*. This could be due to overall effect of DksA on global transcription or by some other mechanism(s), which is yet to be elucidated. In summary, it appears that both RelV and DksA are fascinating proteins and further studies are needed to understand their role in fine tuning the regulation of gene expression in both pathogenic and non pathogenic organisms.
